# An Optimized, Data Distribution Service-Based Solution for Reliable Data Exchange Among Autonomous Underwater Vehicles

**DOI:** 10.3390/s17081802

**Published:** 2017-08-05

**Authors:** Jesús Rodríguez-Molina, Sonia Bilbao, Belén Martínez, Mirgita Frasheri, Baran Cürüklü

**Affiliations:** 1 Research Center on Software Technologies and Multimedia Systems for Sustainability (Centro de Investigación en Tecnologías Software y Sistemas Multimedia Para la Sostenibilidad—CITSEM), Campus Sur UPM, Ctra. Valencia, Km 7, Madrid 28031, Spain; 2 TECNALIA, Parque Tecnológico de Bizkaia, C/Geldo, Edificio 700, 48160 Derio, Bizkaia, Spain; sonia.bilbao@tecnalia.com (S.B.); belen.martinez@tecnalia.com (B.M.); 3 School of Innovation Design and Technology, Mälardalen University, SE-721 23 Västerås, Sweden; mirgita.frasheri@mdh.se (M.F.); baran.curuklu@mdh.se (B.C.)

**Keywords:** autonomous underwater vehicles, cyber-physical systems, middleware

## Abstract

Major challenges are presented when managing a large number of heterogeneous vehicles that have to communicate underwater in order to complete a global mission in a cooperative manner. In this kind of application domain, sending data through the environment presents issues that surpass the ones found in other overwater, distributed, cyber-physical systems (i.e., low bandwidth, unreliable transport medium, data representation and hardware high heterogeneity). This manuscript presents a Publish/Subscribe-based semantic middleware solution for unreliable scenarios and vehicle interoperability across cooperative and heterogeneous autonomous vehicles. The middleware relies on different iterations of the Data Distribution Service (DDS) software standard and their combined work between autonomous maritime vehicles and a control entity. It also uses several components with different functionalities deemed as mandatory for a semantic middleware architecture oriented to maritime operations (device and service registration, context awareness, access to the application layer) where other technologies are also interweaved with middleware (wireless communications, acoustic networks). Implementation details and test results, both in a laboratory and a deployment scenario, have been provided as a way to assess the quality of the system and its satisfactory performance.

## 1. Introduction

Several types of underwater operations play an essential role for many players in the maritime sector. These operations are associated with high costs and, more importantly, high risks due to the fact that human resources (divers) are almost exclusively used. The importance of employing alternative solutions to divers in such operations has been the ultimate goal in this application domain for many decades. In certain cases, Autonomous Underwater Vehicles (AUVs) provide a plausible solution to this problem. By means of technological advancements, future AUVs will most likely be equivalent or superior to human divers regarding the complexity of the operations they can carry out. Although considerable amount of efforts must be made until this vision is realized, today AUVs provide a plausible solution to those missions associated with high costs as well as hazards, especially the ones related to endangering human lives.

In addition to that, the possibility of deploying a set of collaborating AUVs rather than one or even a few scattered units opens up new possibilities. Improving quality and safety for missions, such as subsea mapping, as well as emergence of new missions which require context awareness and collaboration, can be tackled in a better way with several AUVs. As mentioned before, there are a number of challenges associated with development of advanced AUVs. Specifically, hardware interoperability is a major issue at the data level, as the autonomous vehicles supposed to interact with each other may not be able to do so if they send information that cannot be comprehended by a vehicle not manufactured by the same company.

This manuscript puts forward a system aimed to solve these issues (information exchange in constrained environments, hardware interoperability among vehicles from different manufacturers, different data formats representations, etc.) through the usage of middleware for maritime vehicles. By using this approach, it will be possible to overcome all those difficulties and guarantee data transfer among the elements implied in the deployment.

### 1.1. The Need for Cooperation Among Autonomous Underwater Vehicles

Cooperation among AUVs may significantly improve the execution of maritime and offshore operations and missions. Multiple AUVs can work as a team minimizing the need for divers in dangerous missions, reducing the need of costly equipment, saving energy and allowing the achievement of mission objectives of higher complexity than the ones that would be attainable with a single vehicle. There are multiple scenarios where cooperation is useful, such as AUV navigation, ocean surveying or seabed mapping. In the first case, as the cost of small AUVs has significantly decreased, so if maritime vehicle fleets are used, expensive sensors previously required for precise AUV navigation will no longer be necessary in all vehicles, as navigation sensors need to be installed only on one AUV that serves as the guiding vehicle for the fleet [1].

In addition to this, cooperation is being used in several real applications. For example, the WiMUST project uses a team of underwater vehicles acting as intelligent sensing and communicating nodes of a reconfigurable moving acoustic network [2]. Also, mapping the seabed is a vital operation with great importance to industrial, military or biological applications. Unfortunately, due to the imaging geometry of the mapping devices, a map obtained by one single vehicle may not depict the seabed topology correctly. This is why a solution based on map fusion and vehicle cooperation is proposed in the SWARMs project [3].

### 1.2. Middleware and Distributed Systems

A major challenge for cooperation among distributed or cyber-physical systems (CPSs) with different kinds of hardware is the interoperability of the deployed devices. As far as underwater and maritime robotics are concerned, this means that AUVs, Autonomous Surface Vehicles (ASVs), Unmanned Surface Vessels (USVs) or Remotely Operated underwater Vehicles (ROVs) will have to find ways to integrate their heterogeneous capabilities to be able to participate in a mission with a common objective. In this manuscript, autonomous vehicles will always fall within one of the different four categories: AUV, ASV, USV or ROV. AUVs and other types of vehicles belong to different vendors and are designed and manufactured differently, based on either proprietary or open source solutions. Thus, in most cases these vehicles lack the ability to communicate, and hence collaborate with each other, due to different information formats and features that they may use.

Fortunately, many of these issues can be solved by means of a software layer commonly referred to as middleware. Middleware is the software layer used in distributed systems to hide the underlying complexity and heterogeneity of hardware so that a homogeneous-looking set of facilities, commonly provided as an API, will be provided to the higher, more application-based layers. Although its first apparition and usage was documented back in 1968 [4], it later became increasingly popular as a way to guarantee interoperability between legacy and new systems, and it is nowadays one of the most common solutions to address interoperability regarding the Internet of Things (IoT) [5]. Its importance has been recognized by a myriad of different entities for many different purposes, like application implementations or building an environment for cooperative developments. As an example of this latter aspect, the European-driven middleware platform known as FIWARE aims to create a platform where applications can be developed and integrated in a large distributed system by means of the Application Programming Interface that is offered [6]. Middleware can also be enhanced with semantic capabilities which will enrich the information provided by the devices present in a deployment and aid in their integration by offering a common way to define concepts [7].

**Figure 1:**
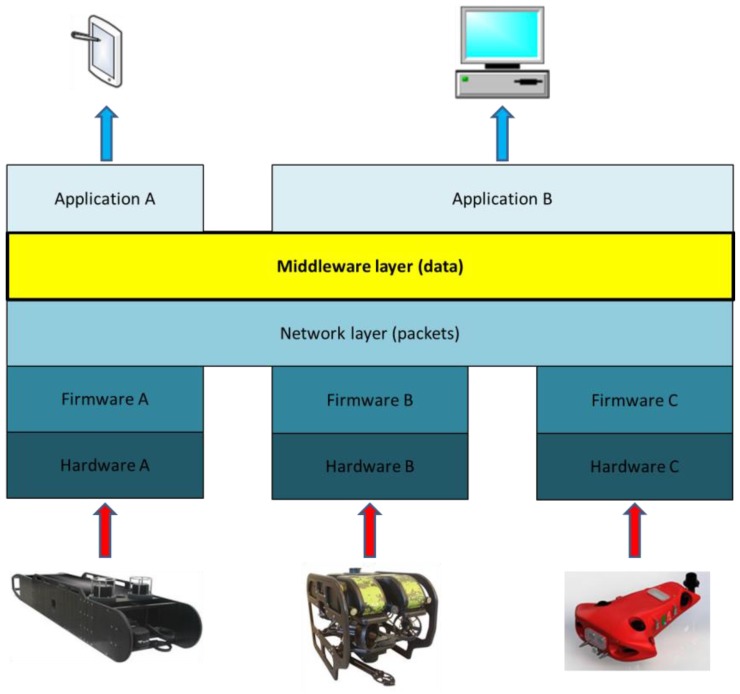
Location of middleware in a scenario with AUVs.

The proposed middleware architecture will work as in any other development classified as a distributed system or a CPS. With regards to this application domain, the need to enable interoperability in heterogeneous robots used in underwater or open sea missions will be dealt with by middleware. Data will also be accessed through the higher levels in the architecture, e.g., by a human operator through a Graphical User Interface (GUI). Note that the middleware among AUVs will hide any issue regarding hardware distribution and will give to the human operator the feeling of working with a local system, as shown in Figure 1.

### 1.3. Interoperability Applied to Constrained Environments

Unlike many environments where systems using different kinds of devices are present and physical transmission of bits can be done in a reliable way with plenty of resources, underwater communications present major issues:

(1) *Unreliable environment*. The most optimized solution for underwater transmission is acoustic waves. In this environment any kind of wireless, regular communication would be unfeasible to transmit with the existing technology. Furthermore, due to the nature of the environment where data are transmitted, information cannot be transferred in a reliable manner and chances of losing data are higher than in wireless, Over-The-Air transmissions (and even higher than in a cabled environment). Underwater acoustic communications are strongly influenced by path loss, ambient channel noise, multi-path, Doppler spread, severe attenuation, high and variable propagation delay, high bit error rates and temporary losses of connectivity. Acoustic signals are much slower than radio waves, and signal propagation is affected by refraction, absorption, high reflection and scattering through the water. Also, attenuation is greater in water than in air [8,9,10,11]. Therefore, mechanisms that optimize data transmissions at higher levels are required, since a Client/Server paradigm may be prone to issues not easy to solve, e.g., resending data due to transmission errors will put strain on the already constrained environment.
(2) *Bandwidth constraints*. All the previously described factors determine the temporal and spatial variability of the acoustic channels, and make the available bandwidth of the underwater acoustic channel limited and dramatically dependent on both range and frequency. Long-range systems that operate over several tens of kilometers may have a bandwidth of only a few kHz, while a short-range system operating over several tens of meters may have more than a hundred kHz bandwidth. In both cases these factors lead to low bit rates [5]. This poses serious challenges for the transmission of a significant amount of bits during a reduced period of time.
(3) *Hardware constraints*. AUVs are supposed to operate in harsh environments. The most critical factor here is the ambient pressure, which increases at a rate of roughly 1 bar for each 10 m. Thus, any construction that will operate in this environment must meet rigorous requirements. In addition, motion speed and battery time are affected negatively, which has a greater impact in this kind of application domain (where motion is done in three dimensions or an expensive robot can be lost if it runs out of energy) than in others. Obviously, both factors influence the communication possibilities of an AUV.

### 1.4. Paper Structure

This paper is structured as follows: an Introduction with the main topics of the manuscript has already been included. Section 2 deals with the related works that have been done regarding middleware solutions in this application domain, along with the open issues and challenges that have been found. Section 3 describes the solution that has been designed and implemented in the SWARMs project; to the best of the authors’ knowledge, this is the best possible development done so far in terms of service availability and distribution. Section 4 comprehends the testing activities carried out in order to evaluate the performance of the proposed solution. Section 5 involves conclusions learned from the deployment and future work. Acknowledgements and References have been included as the last part of the manuscript.

## 2. Related Works

The amount of related work as far as middleware solutions for constrained environments are concerned is rather large. However, the proportion of those solutions that participate in the application domain of maritime and subsea environments is overall scarce, so their applicability for AUVs and similar vehicles (ASVs, USVs, ROVs, etc.) can be regarded as limited at best and completely unsuitable at worst.

### 2.1. Cooperatively Mapping of the Underwater Acoustic Channel by Robot Swarms

Wu et al. described how swarms of underwater robots can be used to generate spatial maps dependent on underwater acoustic communications channels [12]. The authors of this manuscript describe how the channel for the acoustic network has been modelled and identified; a path delay channel model is used for channel identification and modelling. The map making procedures are carried out as a way to find out about the temporal and spatial distribution of unknown quantities. The problem formulation has been dealt with by creating spatial distributions in an application scenario that comprehends an application where a group of autonomous underwater robots are sent to build a network with a mothership in the center. Lastly, map construction is carried out with the measurements that were collected though the trajectories of the underwater vehicles. Simulation tools that were developed to test the proposal are: (a) swarm robot control; (b) channel identification; (c) a spatial-variant ocean environment; and (d) acoustic channel modelling.

All in all, the proposal is fully aware of the multiple challenges that must be faced when transferring information in an underwater communication channel, like multipath effect, limited bandwidth or the large spread originated from the Doppler Effect. The idea of making use of a collection of autonomous vehicles is in line with the works that are planned by the authors of this manuscript. Unfortunately, this paper does not mention how information can be transmitted at the data level or the services that can be provided for the applications, as the main focus on the paper is located one level below this kind of challenges. Furthermore, its scope is limited to the objective of undersea mapping, so there is little information about other services or facilities that can be provided by the system (vehicle registration, semantic capabilities, context awareness, etc.).

### 2.2. Goby3

Schneider claimed in [13] that an open source middleware can be provided in order to offer nested communications among autonomous marine vehicles. One noteworthy contribution of this proposal is its intention of making decisions as close to the source of data as possible so as to avoid an excessive load of traffic. In a more specific way, the proposal establishes a hierarchy of nested communications that defines an interthread level (communications among different threads of the same process), interprocess level (likewise as the previous case but among processes), intervehicle level (data transfers among vehicles) and intersquadron level (data transmitted and received among different groups of autonomous vehicles). A Publish/Subscribe scheme is also used as a way to establish communications between different participants in a deployment of maritime autonomous vehicles. Overall, whenever there are data to be sent involving one entity of the ones defined in the proposal (threads, processes, vehicles and squadrons), it will be done according to the interaction made by each of the Forwarder (used to receive updates about subscriptions and send information about publications) and Portal (use for communications among higher entity levels) implementations in the described levels, with the exception of the layer used for interthread communications, which only has one implemented class called InterProcessTransporter.

The solution that is presented here makes use of a Publish/Subscribe paradigm, which seems as the most efficient way to transfer data among two different entities taking part in an unreliable and constrained environment like in underwater communications. Unfortunately, the middleware solution presented has scarce information about the services that are contained, and does not aim to become an architecture that can provide other functionalities like semantic capabilities.

### 2.3. MAGNA: Middleware for Dynamic and Resource Constrained Sensor Networks

Sinha and Chaczko put forward their solution for dynamic, resource-constrained sensor networks [14]. The authors claim that, apart from typical middleware functionalities (lookup, request/response, discovery, etc.) this solution also comprises a framework divided in three different key bodies related to equally key aspects: the MAGNA Society (an underlying framework for security and communication management, as well as any entropy resulting from the operations of the system), the MAGNA Community (used to gather resources devoted to solve a common task) and the MAGNA Council (the governing body among a plethora of communities in a society. MAGNA has been designed and implemented taking what the authors refer to as the Banking Model approach: a director, branch manager, task manager, broker and teller have been included as the main actors of the system.

Overall, the main issue here is that a Wireless Sensor Network is very different from the kind of environment that is used in this manuscript. While there are several features that have a common scope (deployment of sensors as a distributed system, constrained bandwidth to send and receive information, etc.), the kind of issues present in an underwater environment (unreliable means of transmission, “nodes” moving from their location) are not accurately reflected in a sensor network. Furthermore, the services that can be offered by the middleware solution (semantic enhancement of information, security, context-awareness, etc.) are not described in the paper. Nevertheless, the concept of having parts of the system aware of the relevant blocks of information has been taken into account in the proposal described in this manuscript, as context awareness is a pivotal part of it. This feature has also been included in the original proposal of the manuscript.

### 2.4. Middleware Specialization for Memory-Constrained Networked Embedded Systems

Subramonian et al. offered a middleware solution applied to embedded systems deployed as a network [15]. The main idea is describing the performance of an Object Request Broker (ORB) conceived for real-time, networked systems that has been called nORB. One of the main concerns of this proposal is keeping a memory footprint as small as possible. In order to do so, reducing the middleware footprint in the sensor and actuator nodes has been set as a challenge for the proposal. Some other aspects about the design and development of nORB are provided by the authors, e.g., the proposal was designed having a bottom-up perspective in mind so that just the features that were needed would be included. Among the trade-offs that a user would have to deal with, decreasing the number of fields in the header used or having a worse real-time performance under a large number of operations are mentioned. The authors claim that footprint measurements show an additional overhead of 191 KB for nORB, which is rather low compared to the other brokers included in the study. Lastly, the results obtained from the evaluation of the proposal show that features like performance results, lookup and dispatching or cycle times are comparatively satisfactory, as the other two studied brokers (TAO and ACE) often fall behind.

In the end, the proposal shows a remarkable effort in reducing the amount of memory required for middleware to run over hardware. However, it seems hard to be ported to a system based on autonomous maritime vehicles, as the hardware where middleware would be used is usually less constrained than networked sensors and actuators. Furthermore, the medium of data transmission in this paper is not as challenging as underwater communications, and the fact that the proposal mostly deals with the implementation of a lightweight broker makes it offer few services.

### 2.5. STANAG 4586: Standard Interfaces of UAV Control System (UCS) for NATO UAV Interoperability

This related work is a North Atlantic Treaty Organization (NATO) standard used for interoperability among Unmanned Aerial Vehicles (UAVs) [16]. Monteiro also describes how these vehicles make use of interfaces in order to provide interoperability for them when they have to combine their efforts [17]. This solution describes five different levels for interoperability: level 1 (used for indirect transmission and reception of sensor information and associated metadata), level 2 (reception of sensor product data and the associated metadata that belongs to the aerial vehicle),level 3 (utilized to control and monitor the UAV payload, unless the level has been specified to have just a monitor-only solution), level 4 (control and monitoring of most of the operations of the UAV itself) and level 5 (monitoring and controlling the UAV launch and recovery operations). The standard offers a UAV Control System (UCS) architecture with several software components: Vehicle and Control Data Terminals (VDT and CDT) for connectivity at the data link level, interfaces for Human/Computer interaction as well as for command and control purposes), a core for the UAV Control System and a Vehicle Specific Module (VSM, it interacts with parties like the operator and the launch and recovery system) are among the most prominent ones.

The solution that is described in this standard, though, is clearly focused on Unmanned Aerial Vehicles like drones. In addition to that, there is no information in the standard about how the software components required for services are contained. Nevertheless, the issues faced by the developers of this standard resemble the ones found in this environment (it is explicitly claimed that “This diversity leads to an increased level of difficulty in terms of guaranteed interoperability in teams of heterogeneous vehicles” in [17]). In the middleware proposal that has been developed, VSM, VDTs and CDTs have also been included, as their functionalities mimic the ones described in this related work.

### 2.6. Dynamoth

The authors of this proposal [18] put forward their own view for an interoperability solution in autonomous maritime vehicles, which favors topic-based Publish/Subscribe communications over content-based ones. As studied in other proposals, Dynamoth also relies on a Publish/Subscribe communication paradigm to deliver messages to the entities that have subscribed to a specific topic.; the authors justify its usage by claiming that it can decouple the entities that produce content divided in topics of interest (publishers) from the ones consuming it (subscribers) according to the topic that was previously defined. The proposal is also mentioned to be scalable and cloud-based; the part outside the cloud contains all the clients that integrated in the system. The part inside the cloud has been kept for independent Publish/Subscribe servers. In addition to this, the proposal also offers a hierarchical load balancer that operates at two different levels. One level has been referred to as the system-level and the other one is located at the channel-level. The system level enables the distribution of channels among several Publish/Subscribe servers. The load of information that is managed by each of the individual servers can be changed dynamically, to the point of being able to remove or add Publish/Subscribe servers depending on the requirements of the deployment.

It can be said about this middleware architecture that offers concepts and ideas consistent with the developments carried out in the SWARMs project (decentralized middleware solution, Publish/Subscribe paradigm). Unfortunately, it has been oriented to cloud environments that are hardly related to the application domain that is dealt with in this manuscript, so its applicability for a maritime environment, is reduced.

### 2.7. MOSDEN

In this case, this proposal is aimed at offering a middleware for mobile devices with significant constrains [19]. One of the prominent features of this proposal is its conception as a plugin-based architecture with the aim of supporting three different requirements: scalability (the proposal is claimed to be able to support virtually any available sensor in the world), usability (no programming abilities are required by the users once the plugin of their choice for a specific service) and community-based development (software released will be done so as an open source piece of work so that a growing community will engage in enhancement and extension activities). The middleware architecture has been designed as a layered effort with several levels: information is expected to be obtained from sensors via plugins (where each of the plugins is compatible with a specific sensor) that will collect the information. Plugins are a part of the content of a higher level entity called a wrapper. Several wrappers will be dependent on a virtual sensor. Above those levels, a virtual sensor manager has been designed with the idea of containing several required facilities for the correct performance of the whole middleware (namely, a life cycle manager and an input stream manager containing a Stream Quality manager at the same time), along with another layer used for storage.

The idea of providing a middleware architecture divided in plugins as its main software component unit is appealing in the sense that these components become easy to port from one part of the system to another one. The main issue with this proposal is that it has not been conceived for autonomous maritime vehicles, so as it happens with other ones, its adaptability to the application domain described in this paper is dubious: an AUV or an ASV are very different from the devices that are usually found in an IoT-based deployment.

### 2.8. Distributed Underwater Acoustic Communication Network Simulator

The authors of this proposal [20] studied and emphasized the importance of underwater communication simulations as a way to guarantee the feasibility of a proposed solution. As mentioned in this work, real-world experiments, even when they are controlled environments, are highly costly. Thus, in order to address this issue a novel Distributed Underwater Acoustic Communication Network Simulator (DUACNS) is proposed. This simulator is based on a CORBA (Common Object Request Broker) middleware. As main features, this simulation environment allows modulation and demodulation operations, as well as synthesizing received signals (based on the Bellhop ray tracing channel model). This simulation environment has three main features: firstly, a CORBA middleware, which provides a global time reference for all computers in a simulation and a cross-layer message exchange mechanism for information publishing and subscribing. Secondly, a software-defined modem based on Multi-carrier MFSK, which provides physical layer support, has also been included in the proposal. Lastly, synchronization based on the BELLHOP ray tracing model and 3D network topology is also present.

All in all, while the proposal addresses issues that are common for distributed systems and offers a reliable framework (based on CORBA) for data interchange, its adaptability to an environment where autonomous maritime vehicles are involved in seems questionable, as the proposal seems to focus on the characteristics of the acoustic communications, rather than on the software components or the facilities that are provided for the system where it is included.

### 2.9. MOOS Middleware and Node Adaptivity in Underwater Sensor Networks

In [21] a FP7 project, Underwater Acoustic Network (UAN), is presented. This project assumes that protection of critical infrastructures off-shore and along the coastline requires a multi sensor network, connected to a command and control (C2) center. The project focuses on underwater communication, using mobile nodes through autonomous underwater vehicles. The aim is to enable a communication channel by moving the autonomous underwater vehicles when some of the nodes in the network are moved around (e.g., nodes located on surface vehicles or other underwater vehicles). A middleware specification, Intervehicle Secure Mission Oriented Operating Suite (IS-MOOS) Publish/Subscribe system, is proposed as well in order to address issues related secure underwater communication. This is a Publish/Subscribe system for inter-process communication (IPC), which supports dynamic, asynchronous, many-to-many distributed communication. This work shows that using mobile nodes, in this case underwater vehicles, and the network can adapt to real-world problems, (for example, oceanic variations).

This proposal puts forward several ideas that are most appealing for a middleware architecture used for underwater communications, such as the addition of an explicit layer of middleware and a Publish/Subscribe paradigm for communications. Unlike some others that have been included, the focus of research here is the coordination of Autonomous Underwater Vehicles, which is strongly aligned with the kind of hardware expected to be used in the proposal for hardware interoperability that has been presented in this manuscript. However, the model that it seems to follow is still too focused on networking and mobile nodes, rather than hardware interoperability and services to be offered to the application layer.

### 2.10. Marine Robotic System of Self-Organizing, Logically Linked Physical Nodes (MORPH)

The main goal of this project implied having a collection of separated mobile robot modules that relied on virtual links to interconnect each module and share information among them [22], so that there would be a virtual MORPH Supra-Vehicle (MSV) that would make use of all the combined resources of the spatially separated components. Among the main challenges that the project intended to tackle were negative slopes (which often result in hard detection of single vehicles), adaptive sensor placement and environmental modeling for complex environments. The most relevant part of the MORPH project, considering the proposal that is put forward, is the interconnectivity that was achieved by means of the acoustic network deployed with modems manufactured by Evologics GmbH (Berlin, Germany). They were used as a way to establish communications at the network level, as well as making use of Ultra Short Baseline technology (USBL) for positioning. In addition to that, the modems involved in the deployment that was made were used to measure thruster noise levels in the vehicles used for communications and a safe range was found to operate with them, as stated in [23].

Despite the deployment that was done covered a level lower than the one that this proposal describes, the work that was been carried out here shares some similarities with the objectives of the proposal, even though they are located at the network layer: the integration of the system used to send network-based packages to several different autonomous vehicles and how to transmit data by means of an acoustic network have been taken into account in the testing activities carried out with the proposal of this manuscript.

### 2.11. Other Related Works

Aside from the proposals that have been reviewed, there are some others that should be taken into account due to the fact that include ideas that, while might not be explicitly related to the scenario of several autonomous maritime vehicles interoperating with each other while negating their own heterogeneity, are worth mentioning because they have been used in a similar context. They have been listed in a more detailed way in [24].

Huxley [25] is aimed to offer a collection of software components that can be ported with ease from one vehicle to another while performing functionalities typical of middleware. This piece of work, though, offers little information about how information can be transferred from one vehicle or another without a centralized entity, or even if there is a procedure to do so via Publish/Subscribe components or any other communication paradigm.

According to its developers, Sunrise has as its main objective creating an Internet of Underwater things [26] were reliable communications and data exchanges are made possible, regardless of the vendors that have manufactured the maritime vehicles. Unfortunately, there is no information about the location of the middleware components that are expected to be installed in each of the vehicles or what the performance of the solution is expected to be.

T-REX is another proposal where the stress is put on underwater vehicles becoming capable of making their own decisions [27]. Its authors claim that they have made use of the sense-plan-act paradigm. Other aspects that are mandatory in this kind of application domain, such as the distribution of information among a plethora of underwater robots or features like context awareness or semantics have not been included in the proposal.

Lastly, TRIDENT [28] targets cooperation among maritime vehicles by providing a solution where an Autonomous Surface Craft and an Intervention Autonomous Underwater Vehicle are deployed and interact with each other. Unlike other proposals, there is a semantic world model framework as a way to integrate several autonomous robots, where the different information sources are integrated seamlessly. Security, though, seems not to be considered.

### 2.12. Open Issues, Challenges and Contributions

The previous proposals have been reviewed with the idea of finding out about how the current state of the art is regarding solutions for constrained environments in Cyber-Physical Systems. Taking into account these aspects, the most important open issues have been found are:

(1) *Interoperability solutions for autonomous maritime vehicles are hard to find.* While there is significant literature regarding how information can be transmitted at layers that use bits or packages as the Protocol Data Units, interoperability among elements in a distributed or Cyber-Physical System applied to higher level layers (session, presentation or application layers, which may be covered by a distributed middleware solution) is almost entirely missing.
(2) *Constrained environments do not necessarily match the challenges to be faced in the environment of the manuscript*. Wireless Sensor Networks, embedded systems, or any environment with severe constrains in the computational capabilities of the participating hardware (or software) elements have challenges that are often different from the ones that are found by underwater vehicles. The main difference between this and other environments is that limitations for the maritime vehicles come from the issues existing for communications due to the transmission medium rather than the hardware itself.
(3) *Services available are scarce, if any*. The services that are included in the solutions that have been described before are not that abundant. What is more, there is usually a lack of definition of the services that should be mandatory to have in this environment (hardware registration, access points to the middleware, hardware abstraction, etc.).

Therefore, as a way to tackle those challenges, the following measures are proposed in order to address the challenges associated with interoperability for a set of deployed AUVs:

(1) *Design and implementation of a distributed platform for the cooperation of CPSs*. A middleware-based architecture that behaves as a distributed system where information can be shared by means of messages containing data has been designed. A fully decentralized solution was discarded, since a somewhat central element is required to contain all the services and Human-to-Computer interfaces that is not reasonable to have installed in the autonomous vehicles, either due to their complexity, or since these vehicles are often based on proprietary solutions that require a tailored interface to interact with the other parts of the system.
(2) *Design and implementation of specific services for underwater robots*. The design of the architecture considers all the services needed by a set of AUVs. It has to be noted that the system consists of a set of heterogeneous (both in technical characteristics and other features such as the vendor or the kind of standards used for information transfers) AUVs that must exchange information among them. To the best of the authors’ knowledge, the architecture components that have been defined are unprecedented in any other related work of this nature.
(3) *Usage of suitable communications paradigms*. Even though there are several suitable paradigms to solve how communications are going to take place (as it has been included in Section 3.1) it is widely considered that a Publish/Subscribe paradigm is the most suitable solution for the application domain of underwater vehicles and their ancillary services.

## 3. Description of the Solution

As it has been stated, there are multiple open issues involving solutions for data exchange in constrained environments for underwater vehicles, most of them involving the lack of specific services available, underdeveloped interoperability solutions or how the proposals deal with data from different environments. Nevertheless, there are also several aspects that must be taken into account. For example, it will be proved in this and the next sections that the Data Distribution Service (DDS) standard, which is largely based on the Publish/Subscribe communication paradigm, has been proven to be a reliable solution for interoperability among different underwater resources, and it is capable of handling the challenges related to unreliable environments, communication channels of poor quality and high latency. In addition to that, the design and implementation of a middleware architecture as a way to guarantee interoperability, scalability and interconnectivity of the existing hardware components when the Protocol Data Units that are interchanged at the data level, has been regarded as the most suitable option for the application domain of distributed and Cyber-Physical Systems. It can be claimed that the following technical and scientific contributions are provided by the middleware architecture described in this manuscript:

(1) *Interoperability among autonomous vehicles*. The different kinds of vehicles to be included in the deployments of the SWARMs project deal with several of the types that have been defined before (AUVs, ROVs). The issues that have been described before regarding performance of heterogeneous hardware from different vendors in a CPS will be dealt with by the middleware, since it will abstract the different hardware components and data formats present in the deployment and offer a common way to access those services from upper layers, regardless of the different ways that have been used to represent data formats. As it will be described in the testing section, several different vehicles were integrated by the semantic middleware architecture during the deployment carried out, both in terms of characteristics (AUVs, ROVs) and manufacturers.
(2) *Interoperability upon heterogeneous networking communications*. The semantic middleware architecture presented in this manuscript receives data both from a wireless network that is receiving information from a regular layered architecture (where data are transferred via antennas at the bit level and Internet Protocol at the layer one) and an acoustic network that retrieved information from an AUV navigating underwater making use of a set of acoustic modems to transfer the information about the vehicle involved in the communications. The semantic middleware architecture effectively abstracts this heterogeneity for the final human operator or the staff operating the Graphical User Interface so that they will be unaware of the origin of the data.
(3) *Inclusion of a specific set of services for middleware architectures for underwater environments*. So far, it has not been made clear the specific software components that are needed for a middleware architecture focused on maritime environments; even on other ones, middleware is often based on ad hoc solutions difficult to port to either other application domains or iterate again in the same ones, thus resulting in a waste of resource each time a new middleware solution has to be created. By having a specific list of components it can be set what is needed for underwater environments and develop it in an easier way. Among the most prominent ones, semantic capabilities, context awareness, the existence of an information model, the capability to add security or Quality of Service (QoS) capabilities have been included in the middleware architecture described in this manuscript. In addition to that, there are other components that have been specifically designed and implemented to abstract the features presented in the acoustic and wireless networks used to collect data from the underwater and overwater environments (namely, the CDT and VDT that were referred to in the STANAG proposal. Their implementation, though, has been tailored from the beginning for the semantic middleware architecture described here).
(4) *Development of a Publish/Subscribe infrastructure for underwater vehicles*. To the best of the authors’ knowledge, it has not been used for information transfer at the data level of a layered architecture in middleware solutions for underwater or maritime vehicles. The implementation works that have been done in the semantic middleware architecture make use of CoreDX, a DDS implementation provided by Twin Oaks, for a Publish/Subscribe Manager installed as part of a Command and Control Station (CCS) used to send commands and receive information resulting from the execution of those commands in the vehicles. However, the Publish/Subscribe Manager is but a component of the architecture, which makes use of a plethora of them that, while dependent on the DDS infrastructure to receive data, do not use the latter technology and have been developed from scratch. As it will be described, there are many other components that use different resources.

Considering these aspects and the open issues that were found during the study of related works, Table 1 summarizes the contributions that are made by the middleware architecture described in this manuscript:

Its characteristics and how it has been used in the environment represented in this paper are described in this section.

### 3.1. Publish/Subscribe Communication Paradigm

There are three main paradigms for distribution middleware technologies application domains similar to the one dealt with in this manuscript: Client/Server (for instance, in fields closely related to the one described in this paper, such as mobile robotics in an area [29]), message transfers (in robotics-based swarms like the one depicted in [30]) and Publish/Subscribe environments (used in distributed systems like Wireless Sensor Networks, as shown in [31]). Client/Server architecture connects servers that store or process data to clients that request and use data. For the application domain of this manuscript, though, this kind of architecture has several disadvantages. Firstly, it is too dependent on the server, so if it goes down due to any malfunction, the system will no longer be available. Even any minor issue at the server side of the communication (temporally increased latency, scheduled updates, etc.) will have a significant impact on the whole structure. Secondly, resulting systems are show little scalability, as they rely on communications that may either overload one single entity (the server itself) or have to be improved with a significant change in hardware (a new piece of equipment or replication of the original Server). Thirdly, a big number of client requests directed to the same server can cause network congestion. Lastly, Client/Server communications are typically built on top of TCP (Transmission Control Protocol) which offers reliable delivery but little control over the delivery of Quality of Service (QoS). For example, it is not possible to specify the number and time of retries for dropped packets. What is more, when information is transmitted underwater the usage of TCP and all its underlying elements cannot be guaranteed, so its applicability for this area of knowledge is jeopardized.

Message passing architectures (often referred to as Message Oriented Middleware) implement queues of messages. Rather than calling a process by its name, the system sends a message and relies on the object to select and execute the appropriate code. This approach facilitates encapsulation by hiding both the internal representation of the object and the underlying distribution, as opposed to the many-to-one Client/Server design. However, this architecture does not support a data-centric model, which is believed by the authors of this manuscript to be the most appropriated way to deal with information interchanges. Additionally, applications need to know where to collect their data. Finally, control over the messaging behaviors or real-time QoS is rarely supported.

Publish/Subscribe technology usually adds a data model to messaging. Under this approach, nodes “subscribe” to the data they want to receive and “publish” the data they want to send. Subscribers can dynamically discover publishers of interest and specify QoS parameters to filter the data to receive. Publishers can also set QoS parameters on the data they produce (e.g., reliability, durability, history settings, late-joining readers, etc.). Thus, this approach is optimal for underwater environments that need to adapt to communication channels of low bandwidth and high latency.

Some of the best-known protocols for Publish/Subscribe implementations are DDS, Java Message Service (JMS), Message Queuing Telemetry Transport (MQTT) and the Advanced Message Queuing Protocol (AMQP). While all of them guarantee message delivery, DDS is assumed to offer the best possible Publish/Subscribe mechanism, as it can provide real-time data exchange, space and time decoupling, tight control over the QoS and complete interoperability between different vendors’ implementations.

### 3.2. DDS, DCPS and RTPS

Data Distribution Service (DDS) is the first open international standard addressing Publish/Subscribe middleware architectures for real-time systems. Developed by the Object Management Group (OMG), this standard is focused on connectivity on the data level that acts as the middleware layer in a distributed system [32]. Unlike Client/Server or Message Oriented Middleware (MOM), which is more oriented to processes or messages, DDS is of data-centric nature. Thus, by prioritizing the information contained in the data, it provides a more efficient transmission of contextual information. It is assumed that this approach allows, (i) the inclusion of contextual information, and (ii) enables developers to codify how and where to share data instead of the code required to send messages.

**Figure 2:**
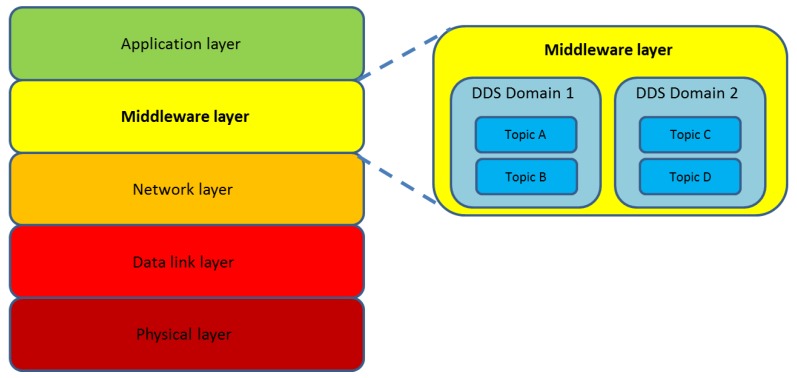
Location of middleware in the system and its DDS components.

Although the IoT is repeatedly mentioned as one of the main areas of interest for the development and application of DDS there are no limits, except for the constrains that may be found in actual deployments, to implement DDS-based works in any other distributed or Cyber-Physical System. It is assumed that DDS is capable of offering a scalable architecture that guarantees high reliability and low latency for data connectivity. While all these features are welcoming under any circumstance, they make DDS more attractive for the application domain of this manuscript, where the challenges imposed by the transmission medium as far as bit and higher levels are concerned make any improvement to reduce latency or make real-time communications desirable. A central idea of DDS is the existence of topics, or data structures that settle the fields and appearance of the information that is transferred from one location to another. Data publishing is done through a topic that is encased in a DDS domain. This domain is separated from other domains and does not share information with them either. In order to exchange information, data-objects contained in topics are utilized, since a topic becomes defined by its name and the data-object is done so by means of key attributes. In addition, data writers that publish content and data readers subscribed to it can filter the information sent and received by time and content. The overall appearance of a DDS deployment, as well as its location in the layered infrastructure that results from applying it to the underwater environment, has been depicted in Figure 2.

In addition to topics, the idea of a global data space is also of major importance, since it implies writing and storing information remotely but gives to the writer the impression that the process is done locally. Interactions with that global data space seem to be done via the Application Programming Interface (API) offered by DDS. However, they actually imply DDS messages being sent to update the involved software infrastructure (stores, remote nodes, etc.). At the same time, the applications store locally what they need just for the required timespan. The global data space is described by the authors of DDS to share data between heterogeneous systems, e.g., cloud applications, embedded and mobile systems, and with low latency.

Another important feature of DDS is the availability of Quality of Service (QoS). QoS specifications include reliability, security or system health. Reliability might also be implemented if required. Dynamic discovery of publishers and subscribers is another feature to be taken into account, as automatic discovery of those makes the underlying hardware easy to include in any deployment. DDS scalable architecture is described as “designed to be scalable from small devices to the cloud and for very large systems” [32]. This approach guarantees that devices of very different features can be integrated in a distributed or Cyber-Physical System. Remote Procedure Calls (RPCs), security and web integration are also central functionalities in this context.

Finally, it is assumed that DDS has been conceived as a scalable architecture for cloud-based deployments. A DDS distributed system is able to contain real-time, QoS and context aware communications. A hierarchy can be established from a networked system point of view, where DDS would be installed in administrative, central, control and machine domains. According to [33], there are several aspects that must be taken into account to fully understand how DDS works:

(1) *Publish/Subscribe messaging*: it is used for the discovery of new services and the management of data flows among the entities involved in a communication.
(2) *Lifecycle awareness*: DDS provides support in information lifecycle awareness for its applications. Features like first and last appearances of data updates in topic instances are, for example, included in this characteristic.
(3) *Relational data modelling*: data are handled as if they were relational databases. DDS relies on structure-related topics and enabling requests tailored in terms of time and content according to the settings used by filters.
(4) *Reliable multicast*: UDP sockets can be used for reliable multicast. While the usefulness of this feature in an underwater scenario is limited, it has been taken into account for testing activities that have been done in order to prove the usefulness of the tested middleware solution.

The main specification of the DDS introduced in its latest version (v1.4, [34]) is a Data-Centric Publish/Subscribe model referred to as DCPS for communication and integration of distributed applications. This solution puts the stress in real-time communications and is described as a model that has become popular in many real-time applications, since it is able to define the concepts of publishers and subscribers according to the context of DDS. The concept of data model, as well as data structures defined by topics and types, are introduced as major ideas in the description of DCPS. However, another aspect of DDS that is of major importance is the existence of ancillary software standards used as a way to offer interoperability for different DDS solutions. The latter refers to Real-Time Publish/Subscribe protocol (RTPS). It is explicitly mentioned in [35] that “This specification defines an interoperability wire protocol for DDS. Its purpose and scope is to ensure that applications based on different vendors’ implementations of DDS can interoperate”, so its objective is guaranteeing interoperability among DDS developments made by different vendors. RTPS can also be described as a wire protocol, in the sense that it establishes mechanisms for communications among remote entities that generate information at one point and send it to another part of a distributed system. The usage of RTPS and DCPS is implicit in the middleware solution that is described in the following section, since they are provided by a DDS development.

### 3.3. Description of a Middleware for Underwater Environments

This section describes the authors’ proposal for a new open-source semantic middleware, which is the central component of a new DDS-based system able to coordinate a swarm of vehicles together with other support vehicles, such as USVs and vessels, to collaboratively achieve a common goal. The middleware solution proposed here is part of an open-source architecture consisting of three main functional components, the Mission Management Tool (MMT), the middleware solution, and the Robot System:

(1) The *MMT* is responsible for generating missions, which are usually composed of (a) tasks; (b) operations required to assign tasks to robots; and (c) operations to supervise the mission. The MMT is located in the CCS, which can be placed on a vessel or onshore during missions. The MMT also contains the Human-Computer Interface (HCI), which allows a human operator to interact with the system, define missions, control each robot, and supervise the evolution of the mission performed by the vehicles.
(2) The *Semantic Middleware* guarantees communication between the MMT and all underwater and overwater vehicles, regardless of type, manufacturer, and capabilities, using buoys or USVs as communication nodes. It uses semantics to infer knowledge from the gathered information. Also, the middleware is the responsible to integrate the DDS communication endpoint on the robot.
(3) The *Robot System* enables a robot to interact and execute the commands provided by the MMT using the Middleware Core. It offers an interface between the underlying functionality of vehicles and the middleware.

**Figure 3:**
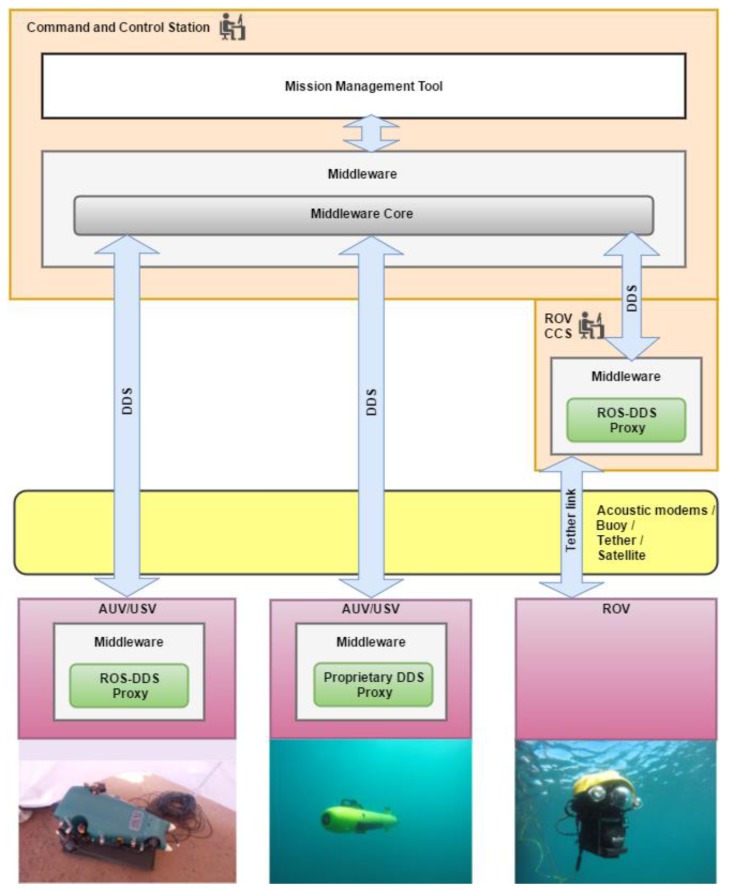
Functional and physical components of the proposed middleware architecture and other related components of the SWARMs.

Functional and physical components of the proposed architecture and the communications links are shown above in Figure 3. Here, the distributed nature of the semantic middleware that has been designed across the different physical components of the maritime vehicles has been highlighted. Note that a *DDS Proxy* module has been placed as the responsible for providing a DDS-compatible communication channel to the Robot System, providing a bridge between the messages used by robots and the DDS messages, thus ensuring that all robots are able to understand that a CCS has been defined. A DDS Proxy is a middleware component that has to be installed in a robot for interoperability between the software components located at the CCS and the robot itself. Being ROS (Robot Operating System) the most common open-source operating system for robots up to now, the middleware provides a ROS-DDS Proxy module whose responsibility is to connect ROS based vehicles to the middleware. Robot Systems with proprietary interfaces (HW/SW solutions for controlling an AUV) could be connected to the middleware to cooperate with other connected vehicles by using a specific ROS Proxy that should be built by the manufacturer. DDS Proxies provide the middleware with the scalability and flexibility needed to integrate heterogeneous Robot Systems. The Semantic Middleware centralizes communications, providing the system the capability to receive, process, and disseminate messages/data between the MMT and the vehicles. The middleware architecture is in charge of mission execution and control, receiving from the CCS the sequence of individual tasks to be accomplished to complete a mission, and sending them to the specific vehicles involved. The middleware also receives feedback from the vehicles involved in the task of a mission and updates the tasks state at the MMT.

Different priorities can be assigned to the different messages exchanged by the middleware depending on the source of the message (e.g., MMT, vehicles) and type of message, e.g., emergency, control, telemetry, etc. Besides, all communications related to command and control data from the swarm of vehicles, such as information that may affect the behavior of the swarm (e.g., battery level of a vehicle) or the planning of missions (e.g., status of an AUV, to determine whether it is occupied or not), must go through the middleware in order to build a shared common information space that enables interoperability and information exchanges between MMT and heterogeneous vehicles in a cost effective manner. This requirement must not prevent vehicles from communicating directly with each other, e.g., two vehicles could share an anti-collision plan (safety related plans), or even forward data from a destination to a source (play the role of a forwarder). The middleware may also support the MMT with useful semantic information for decision-making that should be obtained through semantic queries made to an ontology that has been created to gather and unify information. Contextual awareness is provided through the collection of useful information in order to understand the environment surrounding the available vehicles.

**Figure 4:**
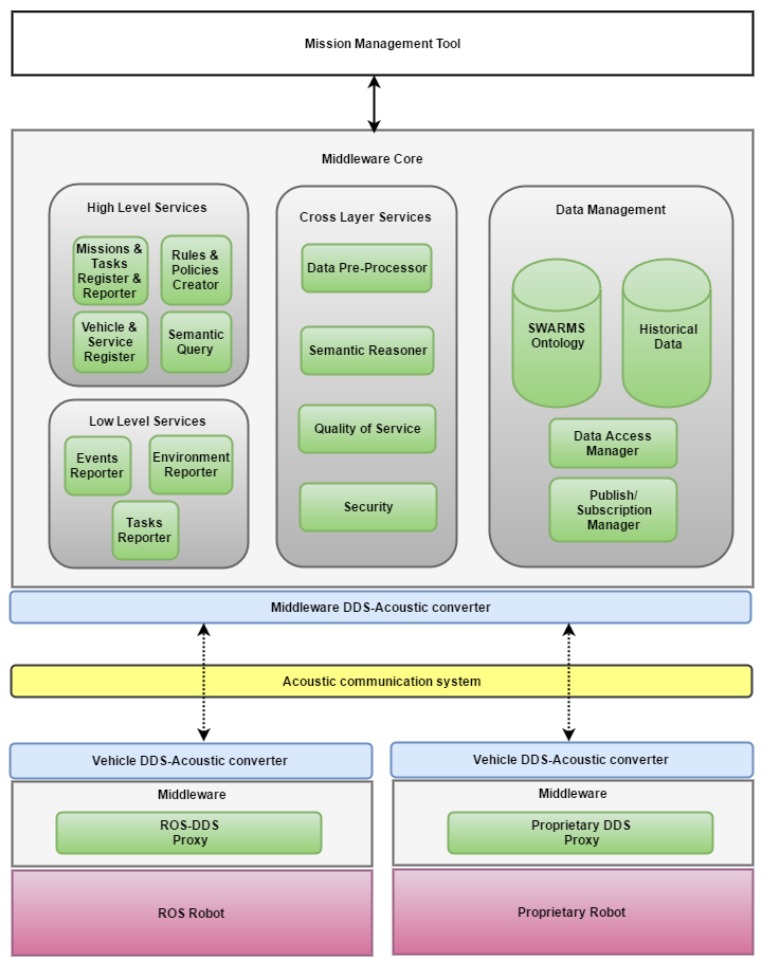
Middleware architecture used in SWARMs.

Figure 4 represents in detail the functional components of SWARMs semantic middleware and its communication links. Note that, regardless of the vehicle type (tethered or untethered) and position (on surface or underwater), taking part in the communication will require different channels. An acoustic channel is used to transmit information to/from untethered vehicles (e.g., AUVs) when they are underwater and an IP channel (e.g., Wi-Fi) both when untethered vehicles are overwater (e.g., AUVs/USVs) and when tethered vehicles (e.g., ROVs) are the receivers or emitters of the communication. For the acoustic case, DDS communications should be translated to an acoustic format. This translation is accomplished by a *DDS-Acoustic Converter*, needed by both the middleware and each of the robots involved. So, there will be a direct communication between the Publish/Subscription Manager of the middleware and the DDS Proxy of the vehicle when using an IP channel, whereas the DDS-Acoustic Converter will act as an intermediary between both ends when using an acoustic channel. The rest of modules of the Semantic Middleware are explained as follows:

(1) *Data management*: it is performed by means of a Data Access Manager, a Publish/Subscription Manager and two repositories, namely the SWARMs Ontology and a Relational Database for historical data:
  ◦ By means of the *SWARMs Ontology*, the middleware defines a Common Information Model to unambiguously represent a swarm of vehicles. The SWARMs ontology consists of a set of sub-ontologies which provide descriptions of different domains of interests to model all information that is necessarily exchanged between any underwater vehicle and architecture component. This ontology is in charge of storing the information obtained from different domains (mission and planning, environment recognition and sensing, communication and networking and robotic vehicles).
  ◦ A *Relational Database* will store historical information related to past events, such as the different positions where a certain vehicle has been operating. Such a kind of information is not convenient to be represented in the ontology given that could be inefficient to access and query. This kind of data is stored in the database each time it has been updated in the ontology.
  ◦ The *Data Access Manager* provides an interface able to insert/retrieve information in the SWARMs ontology or in the relational database. Its functionalities will be invoked when new data are received and have to be included in the database (in case they are historical data that become characterized by its timestamps) or the latest piece of information is included in the SWARMs ontology.
  ◦ The *Publish/Subscription Manager* is a DDS-based component responsible to provide reliable non-blocking communications between the middleware and the underwater vehicles. It is the component responsible for implementing a Publish/Subscribe paradigm for communications, as the semantic middleware architecture will use it to subscribe both the autonomous maritime vehicles and the Command and Control Station to the events that they generate in both sides of the communications. Typically, The Publish/Subscribe Manager generates a collection of DDS topics matched with similar names at other components of the middleware architecture responsible for data transfers (CDT and VDT) so when there is information to transfer it will be done so if received under one of the topics present in both sides of the middleware (due to the fact that the middleware has been distributed to autonomous vehicles as well).
(2) *High Level Services*: these services represent the interface offered by middleware to the MMT. The modules in charge of this responsibility are the Missions and *Task Register and Reporter*, the *Vehicle and Service Register*, the *Semantic Query* and the *Rules and Policies Creator*:
  ◦ The Missions and Task Register and Reporter receives the tasks assigned by the MMT to the robots, and stores this information in the SWARMs ontology. This module is responsible for forwarding the assigned tasks to the corresponding physical robots. As vehicles will follow a Publish/Subscription paradigm, the communication between the Middleware Core and the robots will involve both the Publish/Subscription Manager module and the specific *DDS Proxy* which is able to communicate with a specific vehicle. Note, however, that the middleware will only execute simple tasks and no service composition will be considered, thus any complex task to be carried out by the vehicles will be divided into simple tasks by the MMT. If a specific vehicle needs a more detailed execution, this sub-division of tasks should be implemented by the vehicle itself. Once a task has been executed, this module collects the task status information send by vehicles as a response to the task assigned.
  ◦ The Vehicle and Service Register provides the middleware holistic awareness of all available vehicles and services, so that it will be able to offer real-time and precise information for other modules. This module registers all vehicles with their own features, such as appearance, capabilities, location, status and services provided in the SWARMs ontology. This process is performed every time a new vehicle joins the swarm. The registration process will be initiated from the graphical interface of the MMT.
  ◦ The Semantic Query processes any semantic query made by the MMT. Semantic queries are the mechanism used by the MMT to consult any information from the SWARMs Ontology. The result of the query will involve a semantic piece of information that will be used for information processing at higher level applications (commonly, Graphical User Interfaces) which will contain relevant data related to the autonomous maritime vehicles involved in the area.
  ◦ The Rules and Policies Creator allows the insertion of rules and policies by users. Users-defined rules can be used for reasoning more useful information and knowledge, (for instance, finding out which vehicles’ battery level is above or below a certain threshold). Any semantic query obtains an answer, which is filtered according to the rules defined, however, this filtering is not applied at the moment of calculating the answer: rules are pre-defined and embedded in the ontology, so are executed as soon as new data is inserted in the ontology. This ensures that the responsibility of the ontology is not affected by the rules defined.
(3) *Low Level Services*: these services represent the interface offered by the middleware to the vehicles, in order to monitor the system. The modules in charge of this responsibility are the Tasks Reporter, the Event Reporter and the Environment Reporter:
  ◦ The Tasks Reporter tracks and reports the status of different tasks of a mission. Typically, it will send information to the Mission Tasks Register and Reporter of what has been received from the Publish/Subscribe Manager depending on the request that was executed previously (features of the autonomous vehicle such as battery level, GPS coordinates, etc.).
  ◦ The Event Reporter collects events (task status, alarms, detections, etc.) coming from the vehicles. When they are sent to the higher levels (namely, Mission Management Tool, which is behaving as the application layer of the system) they are displayed for the human operator of the system.
  ◦ The Environment Reporter tracks and periodically reports any environmental data relevant for the mission, to which the MMT has subscribed. As it was done in previous cases, the report will be sent towards the MMT.

All these modules implement a simplistic behavior, collecting and storing specific information in the ontology and informing the MMT later. However, the responsibility to analyze such information and act accordingly is relegated to the MMT.

(4) *Cross Layer Services*: these are services available both to the components belonging to the High Level Layer and the Low Level Layer of the middleware. The modules offering cross layer services to the rest of the modules of the middleware are the Semantic Reasoner, the Data Pre-Processor, the Security module, and finally the QoS module:

◦ The *Semantic Reasoner* is responsible for inferring a higher level of processing or context awareness on the context information stored in the SWARMs ontology. The semantic reasoner processes the gathered context information, and in case semantic rules have been defined, it filters the output previously obtained to achieve all the possible allowable options to support the middleware towards decision making.
◦ The *Data Pre-Processor* allows the middleware to validate data coming from vehicles to ensure that this information is clean and its format is correct, accordingly to the data formats defined and expected, e.g., a temperature must be a numeric value. This module will use the historical data stored in the relational database in order to avoid uncertainties.
◦ The *Security* module covers different kind of security schemes, i.e., data integrity, authentication, authorization, and access identity management. A Public Key Infrastructure (PKI) will be implemented to provide entity and message authentication, and message integrity in overwater communications, while a symmetric key based message signing scheme will provide authentication and integrity in underwater communications.
◦ QoS policies specified by the MMT will be implemented in the designed middleware by means of the QoS features built-in by the DDS protocol.

If the available services present in the architecture are compared to the ones present in the reviewed literature, it can be checked that the semantic middleware architecture put forward in this manuscript has more services than the ones that have been reviewed before. Some of the proposals contain semantic capabilities, hardware abstraction components, security or access for the application layer, but only the one presented as part of the SWARMs project has all the attributes required to perform the functionalities that have been defined as mandatory for semantic middleware architectures in this kind of environment.

## 4. Testing of the Solution

The implementation works carried out for the proposed middleware solution have been tested to ensure its applicability. There have been three different environments where testing activities have been taken and a final collection of tests in the demonstrator used for the SWARMs project. To begin with, the middleware components have been tested locally in a single computer. Secondly, the middleware proposal was assessed with the software components running in two separated laptops that were communicating with each other in a network. Later, the software subsystems that have been developed were included in hardware to perform accurate testing activities. Finally, the software components of the semantic middleware architecture were tested in a deployment with an actual acoustic and wireless network communicating a CCS with an AUV.

### 4.1. Software Components Tests

**Figure 5:**
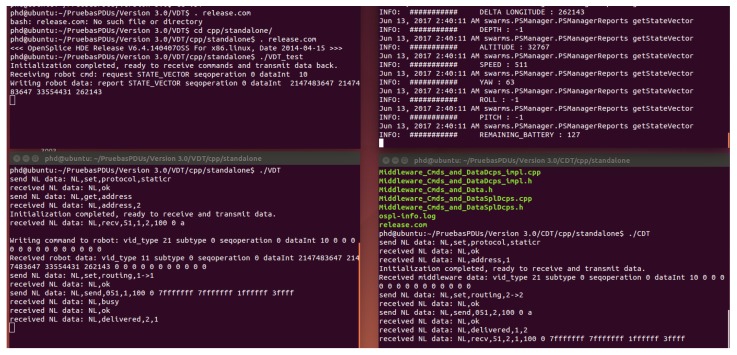
Local tests done with the middleware components.

Local tests were made as a way to perform the operations required to carry on with the implementations works, due to the fact that they had to be regarded as successful before moving forward to more complex scenarios. These local tests were carried out in a machine with an Ubuntu operating system that was capable of handling simultaneously the Publish/Subscribe manager, CDT and VDT software components. Among other features, the connection between CDT and VDT, data transfers among them and the Publish/Subscribe manager sending commands and receiving information were checked. Overall, the performance was as it had been expected, so it was made possible to move the testing to another scenario. The appearance of all the software components being tested locally has been placed in Figure 5.

### 4.2. Simulated AUV-CCS Performed Tests

This section presents the tests that were done to test the Publish/Subscribe communication paradigm used in the middleware architecture. To do so, the configuration and QoS parameters of the DDS distributions were adjusted to successfully send and receive data among participants. It is important to mention that there were three different kinds of tests: compatibility of the features used for the implementation of the middleware solution (namely, compatibility among different vendors and different programming languages), tests aimed to try specific activities for the different scenarios where middleware is involved, and automatic discovery tests were also made as a way to know whether connectivity among distributed software components belonging to different software iterations from the different standard.

#### 4.2.1. Vendor Compatibility, Platform and Language Independency Tests

(1) *Vendor compatibility*: to carry out the tests, two different implementations of the OMG DDSI-RTPS interoperable wire-protocol were used: Vortex OpenSplice DDS Community Edition which is an open-source DDS solution offered by Prismtech [36], and *CoreDX DDS* which is a proprietary implementation offered by Twin Oaks [37]. CoreDX was installed in the Publish/Subscription Manager of the Middleware, while OpenSplice DDS was set up in another piece of equipment to manage all DDS communications in the vehicle (i.e., the ROS-DDS Proxy and the two DDS/Acoustic Converters). CoreDX DDS and OpenSplice DDS were configured to share the same partition (e.g., “swarms”).
(2) *Programming Language compatibility*: to guarantee programming language independency and the progress of developments regardless of the language used to write the software components, all the Middleware Core components were developed in Java, while the components created to be installed in the autonomous maritime vehicles were developed by means of C++.
(3) *Platform compatibility*: the Middleware Core components were installed in a Personal Computer using Windows 8 as the operating system, whereas all the components in the vehicle were installed on ROS and Linux-based hardware. The features of each of the devices have been summarized in Table 2. As it can be seen, neither the hardware requirements are not challenging, nor the middleware components demand a powerful hardware solution or hardware capabilities beyond what is common. Furthermore, the AUV simulated during the tests was done so via virtual machine, yet no problem appeared regarding this feature in terms of performance. This is a major advantage for the project, as autonomous maritime vehicles can be constrained in terms of power and the available energy in their batteries. Specifically, the latter one is meant to be used for their movement and mission tasks rather than for processing operations. Lastly, messages were sent in both directions at very short intervals (every second a new message was sent to the CCS, which is unlikely that will happen in a real world scenario at this high frequency), thus proving that there were no problems regarding latency or data losses.
(4) *Results*: the following compatibility tests were successfully performed:
  - The CoreDX DDS publisher in Java (Windows/Linux) publishes a message in the topic “tasks” and the OpenSplice DDS subscriber in C++ (Linux) receives and reads message.
  - The OpenSplice DDS publisher in C++ (Linux) publishes a message in the topic “status” and the CoreDX DDS subscriber in Java (Windows/Linux) receives and reads message.

The results that were appreciated in those tests were of critical importance, due to the fact that they add an extra flexibility to the implementation works that are being carried out by the partners involved in the project. Due to their own background and know-how, it might be more useful for them to codify by using either C++ or Java, but since both versions are compatible, these preferences do not suppose a significant challenge.

#### 4.2.2. Laboratory Communications Tests for Underwater and Overwater Data Transfers

The goal of these tests was to prove that data can be transferred efficiently from the middleware to an AUV and backwards when the AUV is either overwater or underwater. This means testing both for IP and acoustic channels communications that the middleware: (a) can send data to the vehicle and (b) can receive and understand data from the vehicle and store the information in the SWARMs ontology and database. On the other hand, they are also useful to determine if an AUV (a) can send data to the middleware and (b) can receive and understand requests from the middleware.

**Figure 6:**
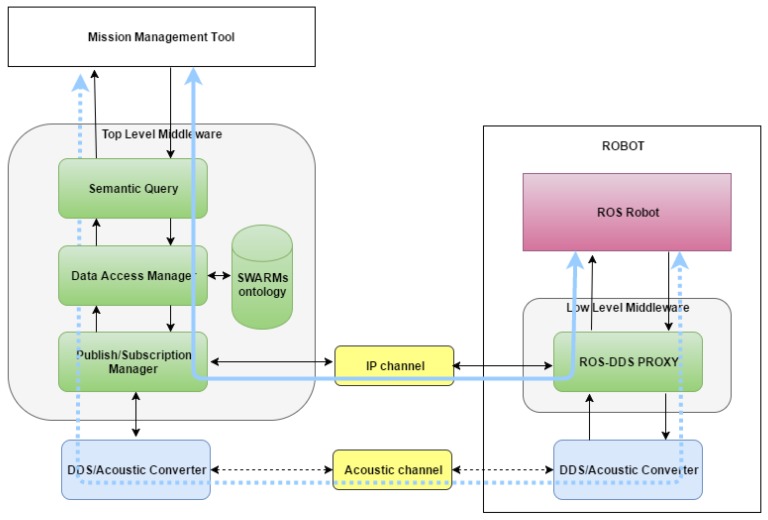
Data transfer architecture.

The complete data transfer process involves the software components depicted in Figure 6. The components related to the middleware are displayed in green, whereas the components related to the communication system are displayed in blue.

Two different communication channels have been tested:

(1) *IP channel*: a WiFi network was established to test the IP communications (displayed in a blue line in Figure 5) between the Middleware and Naiad when it is overwater.
(2) *Acoustic channel*: an acoustic modem was used in order to simulate and test the acoustic communications (displayed in a doted blue line in Figure 5) between the Middleware and the autonomous vehcile when it is underwater.
(3) *Results*: three succesful DDS communication tests were performed:
  - *Acoustic data transfer AUV-Middleware*: the ROS-DDS Proxy publishes the location in the topic “local_data”. The DDS/Acoustic Converter subscribed to this topic reads the data, translates it to the modem acoustic format and transmits it through the acoustic modem. The DDS/Acoustic Converter connected to the middleware converts the data from acoustic to DDS format and publishes the information in the topic “Naiad_data”. The Publish/Subscription Manager subscribed to this topic reads it and sends it to the Data Access Manager so information can be stored in the ontology and transferred to the MMT.
  - *IP data transfer Middleware-AUV*: the Publish/Subscription Manager publishes a task in the topic “MW_tasks”. The ROS-DDS Proxy subscribed to this topic, reads the data, translates it to ROS and sends it to the AUV.

#### 4.2.3. Automatic Discovery Tests

In this case, the goal is to test the dynamic discovery mechanism of DDS, which comes in handy in an unreliable environment such as the underwater scenarios. Dynamic discovery means that it is not necessary to specify in advance the IP address of the participants, as it is expected from them to be anywhere (space decoupling) and that they can start, join and leave in any order and at any time (time decoupling). Thus, for space and time decoupling testing, the vehicle is not subscribed to a topic and has not joined the deployment as a participant. The Publish/Subscription Manager publishes locations in DDS every 5 s. The ROS-DDS Proxy of the vehicle joins as participant and subscribes to the topic. The vehicle successfully receives all the previously published locations.

### 4.3. Testing Activities with NI RoboRIO

**Figure 7:**
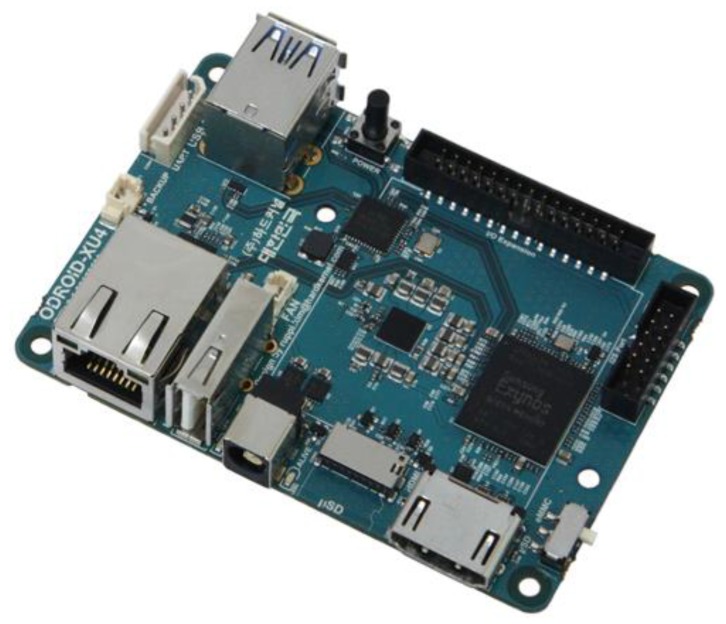
ODROID appearance and interfaces, as depicted in [36].

The testbed used for this activity is a hardware environment that replicates the communication with the Naiad AUV and the proposed middleware. Naiad is a custom-built system designed and manufactured by students and researchers at Mälardalen University since 2013 [38]. The Naiad project aims at developing a fully autonomous system with advanced data processing capabilities on board including stereo vision using a dedicated FPGA. The current Naiad system is powered by NI RoboRIO [39,40]. The rationale behind assuming powerful computing units in the design is related to the limitations found in underwater communications. Since the environment where AUVs are deployed is hostile in terms of power consumption and unreliability of the transmission medium, it is hypothesized that computing power is essential for a fully autonomous system in such an environment in order to collect and pre-process data, infer knowledge from them and finally take decisions similar to a human, i.e., when support from the outside world is absent. The low level components of the middleware are installed on the ODROID Xu4 external device [41]. Its general appearance has been displayed in Figure 7.

**Figure 8:**
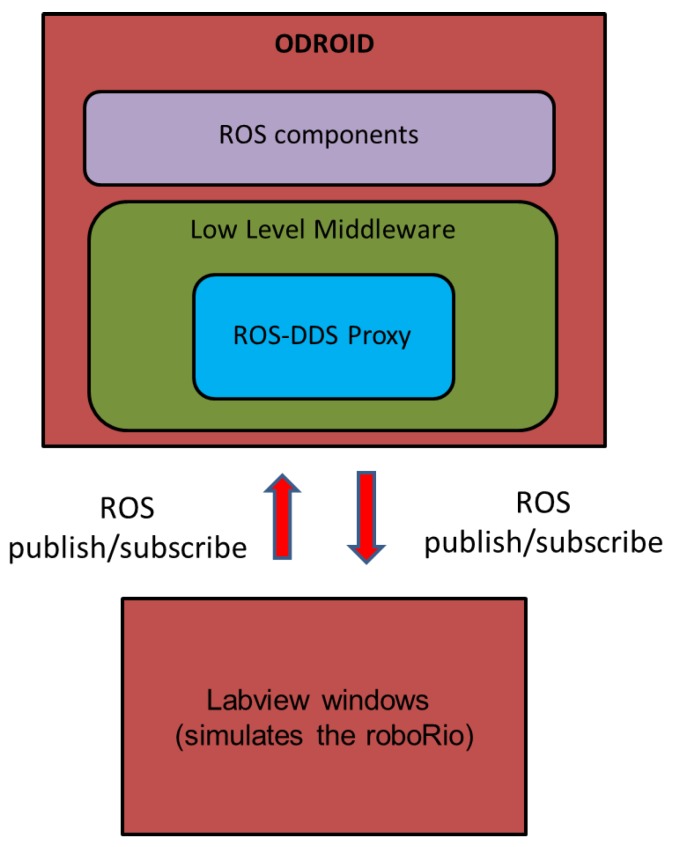
Middleware components used in the tests done with Windows Labview and Odroid.

Beforehand the environment on the ODROID was setup with ROS [42] and OpenSplice DDS [36], which is the C++-based version of DDS that had been tested previously in terms of compatibility. The system on the RoboRIO is being developed using LabVIEW. The communication between the components will use the TCPROS protocol [42], which is a TCP layer specifically developed for services and messages based on ROS. The overall appearance of the components installed was as shown in Figure 8.

**Figure 9:**
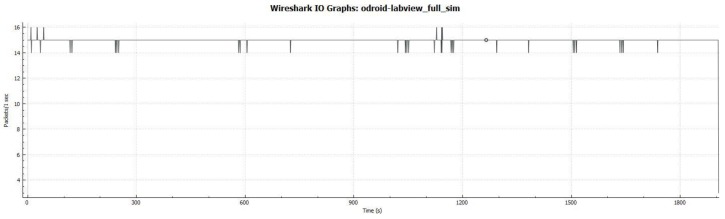
ODROID packet publication, as represented by Wireshark.

Preliminary tests simulate such communication scheme using a LabVIEW system installed on a Windows machine. Specifically, testing has been carried out with the low-level middleware continuously publishing data to the LabVIEW system, while simultaneously subscribes to data produced by the latter. The tests are run for 30 min. The round trip time for the transmission of the packets is calculated using Wireshark [43]. It can be seen in Figure 9 how messages were published at a high rate by the ODROID system, which acts as the AUV in a real scenario publishing information about the required features. Specifically, 15 data packets were published every second, which gives an idea of the publishing capabilities of the system. These tests were useful to show that the Publish/Subscribe mechanisms that have been created are way more robust than the message load they are expected to face. Higher transmission rates were achieved during the testing activities in the demonstrator.

**Figure 10:**
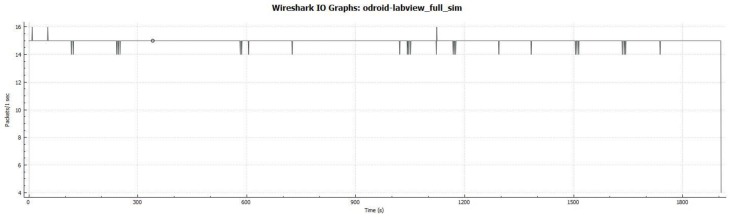
LabVIEW packet publication, as represented by Wireshark.

Furthermore, if the roles are inverted, and the LabVIEW Windows system (which represents the CCS in the Publish/Subscribe communications) is the one publishing messages, its capabilities are the same than the ones that had been previously shown by the ODROID-based system, that is to say, 15 messages were measured as the ones transmitted through the network. The small variations in messages published can be regarded as insignificant due to the fact that the system is capable of publish more amount of information than the one that is expected to be present in an actual deployment. These results have been portrayed in Figure 10.

**Figure 11:**
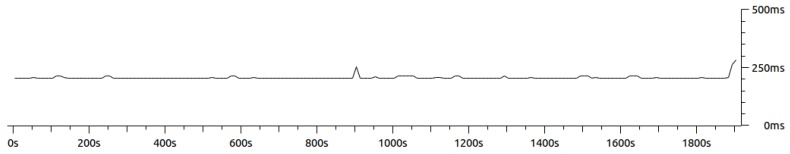
Round Trip Time for data deliveries.

Lastly, measurements were taken regarding the Round Trip Time (RTT) that would take for a message to be delivered and sent back. The results shown in Figure 11, which refer to the same testing activities made to obtain the other graphs, show that the maximum RTT in each delivery was measured as 250 ms, which is acceptable for the kind of system that is being built, since it is an almost imperceptible time for a human end user and data can still be retrieved in an almost-real time fashion so as not to delay any mission where data transmissions are required. Calculation of the RTT is done by Wireshark considering the maximum RTT of the packets that have been delivered in a tic of time, being 10 s long in this case.

It has to be noted that in all these graphs the X axis contains the 30 min used to make the tests, whereas the Y axis is used for each of the variable results.

### 4.4. Testing Activities during the SWARMs Demonstrator

The proposal for middleware architecture that has been described and tested in laboratory-based environments was finally tried on vehicles and facilities during the first demonstrator made possible in the framework of the SWARMs project during July 2017. Among the different tests carried out during the demonstrator, the integration between the semantic middleware architecture and the communications established at the network layer were made with actual pieces of equipment, which were as follows:

(1) An AUV provided by ECA Robotics called A9 [44] used to receive information from the activities that it was carrying out.
(2) Two acoustic modems provided by Evologics GmbH [45] utilized to communicate the A9 with a USV by means of underwater acoustic waves.
(3) A USV tailored for the demonstrator by Leonardo Defence Systems [46] employed to convert and transfer the information received from the acoustic network to the wireless, Over-The-Air one installed by TTI Norte SL [47].
(4) A laptop where the semantic middleware components were up and running. Among those components, the most prominent one for the scope of the manuscript is the Publish/Subscribe Manager, used to send commands to the A9 dealing with the information it was capable of providing.

In the procedures that were undertaken, it was tested how information from a State Vector request could be obtained from the AUV and sent back to the laptop running the P/S Manager. By State Vector request, it is meant a request for information formatted as a message that contains the following relevant data:

(1) An identifier of the vehicle used to receive information (in this case, the A9 from ECA).
(2) A timestamp to show when messages were delivered. Additionally, this piece of information is also useful to know how long it took for a message to be received after a previous one was delivered.
(3) GPS coordinates. They are useful to determine univocally the position of the AUV.
(4) Depth. It determines how deep the AUV is diving.
(5) Altitude. It determines the vertical distance between the AUV and the seabed of the area where it is diving.
(6) Speed. It is used to determine how fast the vehicle is moving through the sea.
(7) Yaw, pitch and roll angles. Those parameters are used to know the manoeuvers being carried out by the vehicle under the water.
(8) Battery level. It is used to know how much energy the AUV has at its disposal.

As far as the length of the information included is concerned, it was formatted as a Protocol Data Unit of the protocol that has been described at [48], so it was guaranteed that the PDU would not be too long and would be challenging to be handled by the acoustic network used to transfer data at a level below the semantic middleware solution. Specifically, the length of the fields that were contained was as follows:

(1) The type of message that was sent was included in the lower 4 bits of the byte that was used to encase both the vehicle identifier and this piece of information.
(2) The subtype of message (since there are many different kinds of reports that can be sent to the C & CS where the P/S Manager is running, a subtype is required to determine the kind of information that is being sent).
(3) The eight pieces of data previously described.

**Figure 12:**
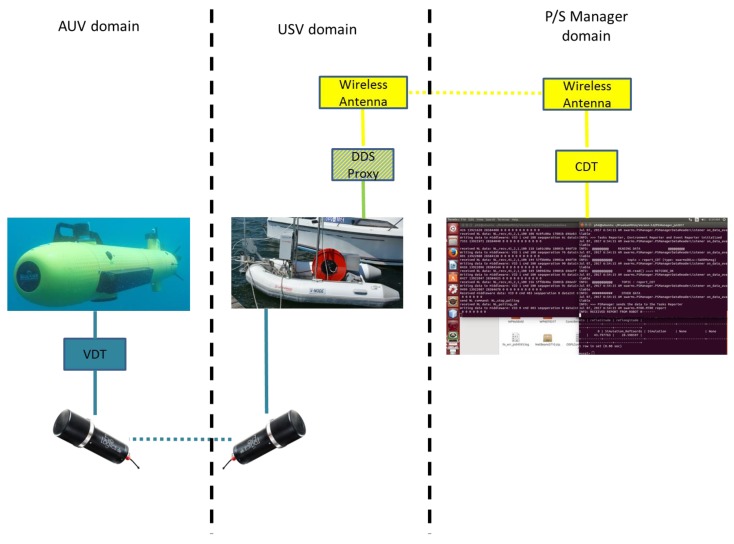
Deployment of elements during testing activities.

The scenario that was created has been summarized in Figure 12. As it has been explained previously, all the elements involved in a holistic deployment for testing activities have been included: (a) the acoustic and wireless networks used to communicate all the hardware and software elements at the network layer; (b) the CDT and VDT used for data formatting between the networking components and the semantic middleware solution; and (c) the hardware devices used by the system (namely, the AUV, the USV and the C & CS where the Publish/Subscribe Manager is running). The capabilities of the piece of equipment used to run the semantic middleware solution were as the equipment used to check vendor compatibility in the first collection of tests. Once the scenario was deployed, measurements were taken to know whether information could be retrieved and the frequency and pace of that information retrieval. The objectives with these testing activities were (a) ensuring that information could be obtained by the system and (b) that it was collected at an acceptable rate. There were some challenges to be faced with these operations: to begin with, the transmission medium for the acoustic waves made not possible guaranteeing a flawless transmission of data all the time. Furthermore, during the testing activities the reliability of that medium of transmission would change frequently and become better or worse depending on the water temperature, how strong the wind blew during a certain moment of the day, etc.

**Figure 13:**
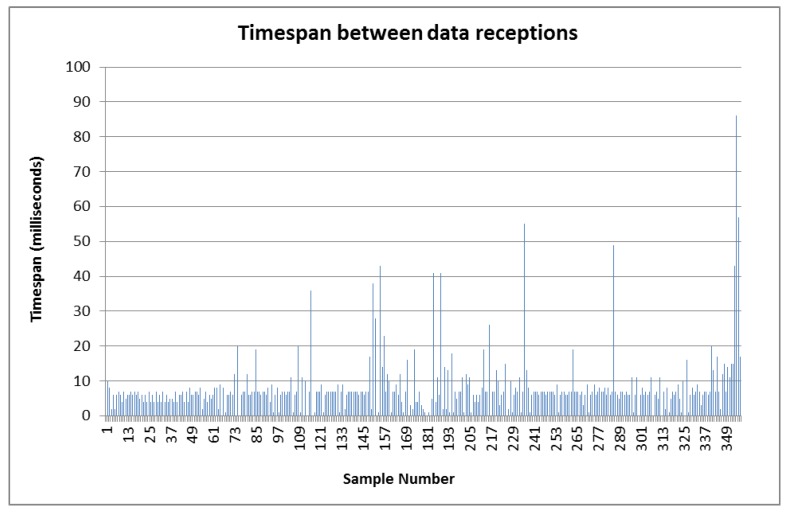
Timespan values between receptions of Status Vector information.

A relevant amount of data was retrieved during the tests performed with the equipment that was deployed. Considering the objectives of the tests, 357 relevant pieces of information were retrieved where information about altitude, depth, speed or remaining battery data were obtained. The time difference among each of the pieces of information retrieved has been displayed in Figure 13.

As it can be seen, the results range from 2 ms (which is the minimum value) to 86 ms (the maximum value). The tests measurements shown here were taken with good weather and water conditions. When they were worse, suboptimal periods of time where data would take much longer to be received (several seconds) happened, but just during rare periods of time (less than 1% in the measurements taken), so they were deemed as not representative of the overall performance of the system. Median value was measured at 7 ms, whereas the obtained average value was 7.882 ms. There are several outlier values that distort the average value obtained and add certain difference with the median value (which can be considered as a more realistic way to assess the performance of the system, as it is not significantly accepted by outlying values), as the median value represents the 88.81% of the average one (and thus is somewhat significantly lower than the average). This can be due to the unpredictability of the medium of transmission of the acoustic waves, since it is the only part of the system with changing parameters. Also, it can be expected that should any of the modems be placed nearby submarine water currents, there could be a significant impact on the placement of the acoustic modems (for example, they could be occasionally tilted by the water current, which would affect how the acoustic waves are transmitted). Nevertheless, the measurements done prove that the semantic middleware solution makes possible retrieving information from the undersea environment without having a negative impact on the time required to transmit and receive data at the communications network.

### 4.5. Discussion and Comparison of the Performance Results

Overall, testing activities can be regarded as satisfactory. The interoperability between different vendors, programming languages and operating systems was guaranteed after the first set of tests done by means of non-robot based machines. Additionally, communications at different layers were proven functional despite all the challenges that can be found in the application domain described by this manuscript. Finally, testing carried out by means of communications with the LabVIEW system conclude that the system can be ported with success to hardware environments like the ones to be expected in Autonomous Underwater Vehicles. These results will be further expanded when the middleware architecture is incorporated to the other robots that are going to be used in the project whenever new deployments are done in further testing activities.

If this proposal is compared to other tested solutions already present, performance results are comparable with them. For example, Phuong Nguyen et al. [49] mention that machines of 4 cores, 3.4 GHz and 8 GB of RAM were used to realize scaling for testing of their middleware solution. Their performance results show that delay remains low when the number of subscriptions gradually increases, which mirrors the capabilities of our systems, capable of handling 15 packets per second in a Publish/Subscribe connection with negligible delays. Additionally, Sven Akkermans et al. [50] deal with Elastic Publish subscribe systems that follow a performance modeling-based approach. According to the authors, they get average times for job executions that are in the range of seconds, which again is aligned with the results obtained in our proposal. However, if compared to other solutions that offer information about their performance, the middleware architecture that has been presented in this manuscript has been developed with underwater environments in mind where Publish/Subscribe systems have either not applied or have been done so to a lesser extent than the components and procedures that are described in this manuscript. Other proposals show similar performance results. For example, in the performance evaluation carried out for the proposal of Publish/Subscribe middleware done by Sven Akkermans et al. [51], it can be seen how the inclusion of a Publish/Subscribe middleware with an underlying IPv6 network for a multicast scenario results in just a small increase of the memory footprint (1.3%) and dynamic memory (4.7%) of the system, with a progressive increase in the use of bandwidth for a higher number of nodes (tests have been made with up to 20 nodes using unicast and multicast solutions). Furthermore, in the study made by Yali Wang et al. [52], it is shown how a Publish/Subscribe architecture oriented to the IoT shows an average delay in the order of seconds when messages are transmitted via IP network. Average loss rates are kept between 0 and 6.41%. Finally, Pablo Picazo-Sanchez et al. study secure Publish/Subscribe protocols for Body Area Networks [53]. Execution of cyphered information is the most important feature treated in this latter scientific paper; setup, encryption and decryption activities increase the amount of time required when the number of attributes gets higher, and then stabilizes at 10–15 of them, but the time required for the execution is never higher than 5 s in the worst case scenario (decryption with 30 attributes).

Overall, even though they show the same kind of performance, the proposal described here has more services and software components integrated in it, along with functionalities that are not present in the other ones. What is more, it has integrated both different kinds of vehicles and manufacturers (an AUV and a USV from different companies) as well as network communications that rely on very different kinds of technology to transfer the data. When all is said and done, the tests that have been done so far show no significant sign of unforeseen events that may jeopardize the system in the way that has been conceived. More importantly, they seem to be able to withstand the significant number of requests that has been used without jeopardizing data delivery or performance results. This is revealing in the sense that the development works that have been done so far can continue in the same direction and there will be little need in modifying extensive parts of the code that has already been created.

## 5. Conclusions and Future Works

This manuscript contains several contributions that, to the best of the authors’ knowledge, have not been matched by the existing literature regarding interoperability for underwater vehicles. A study has been carried out where the software works and tools for interoperability solutions in constrained environments have been assessed, according to their advantages and weaknesses. After the open issues and challenges have been extracted from this study, a solution based on a middleware architecture specifically tailored for autonomous maritime vehicles has been put forward, describing its elements and the reasons to consider that improves the existing state of the art. This solution contains several software components that deal with the most representative functionalities to be expected from middleware (device registration, semantic enhancement of information, requests and responses under a Publish/Subscribe paradigm, etc.). The solution makes use of the capabilities that can be offered from DDS in order to guarantee several features that come in handy for middleware implementation, as well as guaranteeing the interoperability among the different vehicles that have been included in the SWARMs project. Lastly, the implementation works that have been developed have been tested in two different scenarios that prove the feasibility of the proposal described in this manuscript. The testing activities that have been undertaken prove that information can be transferred by means of the middleware solution proposed, which becomes distributed between a partially decentralized element (the Command and Control Station) and the autonomous vehicles that have all the other components of the middleware. When all these aspects are taken into account, it can be said from the middleware solution that has been depicted in this manuscript that it shows promise regarding the integration of the final set of autonomous maritime vehicles that are going to be included in SWARMs. What is more, performance confirms that it will be possible to transfer data through a system of distributed robots in underwater environments with all the features that have been developed for the system.

There are several future works that are going to be made during the following stages of development of the middleware architecture. The middleware components expected to be installed in the robots will be done so in the ones provided by other partners. In case there is any challenge for the integration of the components in the architecture, a go-between solution will be developed in order to integrate the autonomous vehicles unable to have the solution installed by means of an interface that will provide connectivity with all the other parts of the system. Furthermore, the extension of the development activities to all the other software components of the middleware guarantees that all the other features will become fully functional by the end of the project without deviations in its schedule.

## Figures and Tables

**Table 1:** Summarized contributions of the middleware.

Technical Issues Addressed	Actions Taken in the Middleware to Solve the Issue
Interoperability among autonomous vehicles	Hardware abstraction, information model, simple interfaces to access middleware services from higher levels
Lack of specific set of services	Definition of specific set of services for middleware architectures applied to underwater environments
Publish/Subscribe infrastructure for underwater vehicles	Development of a middleware with Publish/Subscribe paradigm for decoupled reception and delivery of messages (better for underwater environments with unreliable transmission medium)

**Table 2:** Hardware features of the pieces of equipment used.

Equipment Features	
Simulated Autonomous Underwater Vehicle	Random Access Memory: 2 Gigabytes. Hard Disk Drive: 20 Gigabytes. Operating system: Ubuntu 14.04 LTS running on VMware Player 4.0.6. Middleware components size: less than 2 Megabytes.
Simulated Command and Control Station	Random Access Memory: 8 Gigabytes. Hard Disk Drive: 1 Terabyte. Operating system: Windows 8. Middleware components size: less than 4 Megabytes.
